# Regulation of Polar Peptidoglycan Biosynthesis by Wag31 Phosphorylation in Mycobacteria

**DOI:** 10.1186/1471-2180-10-327

**Published:** 2010-12-29

**Authors:** Charul Jani, Hyungjin Eoh, Jae Jin Lee, Khozima Hamasha, Moodakare Bheema Sahana, Jeong-Sun Han, Seeta Nyayapathy, Jung-Yeon Lee, Joo-Won Suh, Sang Hee Lee, Steve J Rehse, Dean C Crick, Choong-Min Kang

**Affiliations:** 1 Department of Biological Science, Wayne State University, 5047 Gullen Mall, Detroit, MI 48202, USA; 2 Department of Microbiology, Immunology and Pathology, Colorado State University, 1682 Campus Delivery Ft. Collins, CO 80523, USA; 3 Department of Physics & Astronomy, Wayne State University, Detroit, MI 48201, USA; 4 Department of Biological Sciences, Myongji University, San 38-2 Namdong, Yongin, Gyeonggido, 449-728, Republic of Korea

## Abstract

**Background:**

Sensing and responding to environmental changes is a central aspect of cell division regulation. *Mycobacterium tuberculosis *contains eleven Ser/Thr kinases, two of which, PknA and PknB, are key signaling molecules that regulate cell division/morphology. One substrate of these kinases is Wag31, and we previously showed that partial depletion of Wag31 caused morphological changes indicative of cell wall defects, and that the phosphorylation state of Wag31 affected cell growth in mycobacteria. In the present study, we further characterized the role of the Wag31 phosphorylation in polar peptidoglycan biosynthesis.

**Results:**

We demonstrate that the differential growth among cells expressing different *wag31 *alleles (wild-type, phosphoablative, or phosphomimetic) is caused by, at least in part, dissimilar nascent peptidoglycan biosynthesis. The phosphorylation state of Wag31 is found to be important for protein-protein interactions between the Wag31 molecules, and thus, for its polar localization. Consistent with these results, cells expressing a phosphomimetic *wag31 *allele have a higher enzymatic activity in the peptidoglycan biosynthetic pathway.

**Conclusions:**

The Wag31_Mtb _phosphorylation is a novel molecular mechanism by which Wag31_Mtb _regulates peptidoglycan synthesis and thus, optimal growth in mycobacteria.

## Background

Tuberculosis (TB) is a major health problem with a high mortality worldwide [1]. During the infection, *Mycobacterium tuberculosis *is able to remain dormant in the human host without causing active disease for prolonged periods. Despite the importance of latency in the epidemiology and pathology of TB, it is not clear how *M. tuberculosis *controls the latent state in human host. However, to achieve, maintain, or escape from the latent state, *M. tuberculosis *must carefully regulate cell division by sensing and responding to specific signals in the host environment. To successfully complete this essential process, the *M. tuberculosis *genome contains a wide variety of transcription regulators, surface receptors, and signaling molecules including eleven "eukaryotic-type" Ser/Thr protein kinases (STPKs) [2]. We previously showed that two of these kinases, PknA and PknB, are key components of a signal transduction pathway that regulates cell morphology [3]. One substrate of these kinases we identified is Wag31, a homolog of the cell-division protein DivIVA in other Gram-positive bacteria [4,5].

DivIVA functions in cell division in many Gram-positive bacteria, but the specific roles it plays vary in a species-specific manner. For instance, *Bacillus subtilis *DivIVA has dual functions in this microorganism: it is required for appropriate septum placement by confining the MinCD cell division inhibitory complex at the cell poles in vegetative cells, and it facilitates chromosome segregation by interacting with the *oriC *complex in sporulating cells [6-8]. In contrast, DivIVA in *Streptomyces coelicolor *is essential for hyphal tip growth, and DivIVA homologs in *Corynebacterium glutamicum *and *Brevibacterium lactofermentum *are localized to the cell poles and are required for their polar growth [4,9,10].

We, and others, recently demonstrated that *wag31 *is an essential gene, and that Wag31 is localized at the cell poles in mycobacterial cells [11,12]. We further showed that the partial depletion of Wag31 causes dramatic morphological changes indicative of defects in polar peptidoglycan biosynthesis, and that Wag31 and nascent peptidoglycan biosynthesis co-localize at the cell poles, suggesting an important role of Wag31 in polar peptidoglycan biosynthesis in *Mycobacterium smegmatis *[11]. Finally, expression of phosphomimetic *M. tuberculosis wag31 *(*wag31T73E_Mtb_*) in the *wag31_Msm _*deletion mutant of *M. smegmatis *showed higher growth rate than cells expressing wild-type *wag31_Mtb _*or phosphoablative *wag31T73A_Mtb _*[11]. While Wag31_Mtb _appears to have a role in the protection of mycobacterial cells under stress conditions [13], these observations strongly suggested that Wag31 and its phosphorylation plays a critical role in modulating cell growth through regulating peptidoglycan biosynthesis in mycobacteria.

In the present report, we further characterize the role of Wag31 phosphorylation. We show that the differential growth caused by the expression of different *wag31_Mtb _*alleles (wild-type *wag31_Mtb_*, *wag31T73A_Mtb_*, and *wag31T73E_Mtb_*) is due to, at least in part, dissimilar nascent peptidoglycan biosynthesis. We further show that the phosphorylation state of Wag31 is important for protein-protein interaction between the Wag31_Mtb _molecules, and thus, for its polar localization. In line with these findings, we observe a higher enzymatic activity (MraY and MurG) of peptidoglycan biosynthetic pathway in cells expressing phosphomimetic *wag31T73E_Mtb _*than cells expressing wild-type *wag31_Mtb _*or phosphoablative *wag31T73A_Mtb_*.

## Results

### Phosphorylation of Wag31 affects the polar peptidoglycan biosynthesis in mycobacteria

**Figure 1:**
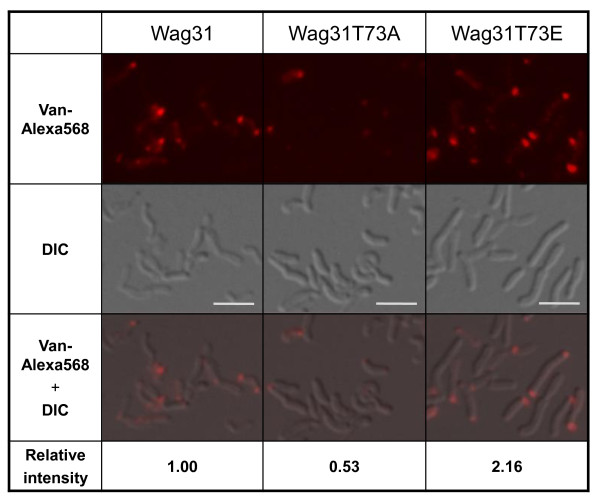
**Effect of Wag31 phosphorylation on nascent peptidoglycan biosynthesis**. *M. smegmatis wag31_Msm _*deletion mutants containing wild-type P_tet_-*wag31_Mtb_*, P_tet_*-wag31T73A_Mtb _*or P_tet_*-wag31T73E_Mtb _*was cultured until mid-log phase and incubated with Van-alexa568 (5 μg ml^-1^) for 20 min at 37°C. Cells were washed with PBS buffer and examined by an Olympus BX51 microscope. To quantify the polar fluorescence intensity, DIC (middle panel) and fluorescence (upper panel) images were superimposed to align cells and fluorescence signals (lower panel), and the average fluorescence density from the poles of approximately 300 cells was determined by using the ImageJ software. Intensity of fluorescence signals relative to that of cells expressing wild-type *gfp-wag31 *is shown. *p*-values for the difference (one-tailed, unpaired t-tests): wild-type Wag31_Mtb _vs. Wag31T73E_Mtb _= 1.1 × 10^-4 ^significant, wild-type Wag31_Mtb _vs. Wag31T73A_Mtb _= 3.3 × 10^-10 ^significant (significant to p < 0.05). bar, 5 μm.

Previously, we constructed a conditional *wag31_Msm _*mutant of *M. smegmatis *to demonstrate that *wag31 *is an essential gene [11]. When the phosphomimetic *wag31 *allele of *M. tuberculosis *(*wag31T73E_Mtb_*), as a sole source of Wag31, was expressed in this mutant, a higher growth rate (mean doubling time, *g *= 4.30 h) was observed than cells expressing wild-type *wag31_Mtb _*(*g *= 4.95 h), and cells expressing the phosphoablative *wag31T73A_Mtb _*allele showed the lowest growth rate (*g *= 5.75 h) [11]. Since Wag31 had been suggested to play a role in polar peptidoglycan biosynthesis [11,12], we tested whether the differential growth phenotype among these strains was due to, at least in part, a difference in peptidoglycan biosynthesis. To investigate this, we cultured those *M. smegmatis wag31_Msm _*deletion mutants expressing *wag31_Mtb _*(KMS41 in Additional file 1 (Table A1), *wag31T73A_Mtb _*(KMS42) or *wag31T73E_Mtb _*(KMS43) until mid-log phase, and stained with Vancomycin-Alexa568 conjugate (Van-Alexa568) to examine by fluorescence microscopy with fixed exposure time and diaphragm aperture settings. Fluorescent vancomycin binds to the terminal D-Ala-D-Ala portion of unincorporated, or incorporated but uncross-linked, peptidoglycan precursors on the cell surface, and thus has been used to identify the sites of nascent peptidoglycan biosynthesis in Gram-positive bacteria [14]. Van-Alexa568 signals from the polar regions of the cells expressing *wag31T73E_Mtb _*was approximately four-fold higher than those expressing *wag31T73A_Mtb _*(Figure 1). Cells expressing the wild-type *wag31_Mtb _*allele showed an intermediate intensity of Van-Alexa568 signals, consistent with its growth phenotype [11]. Thus, this result suggests that the phosphorylation state of Wag31 either regulates polar peptidoglycan biosynthesis, possibly by directly or indirectly affecting enzyme(s) in the peptidoglycan biosynthetic pathway, or affects the level of cross-linking of peptidoglycan leaving non-crosslinked D-Ala-D-Ala.

### Protein-protein interactions and polar localization of Wag31 molecules are affected by phosphorylation

The DivIVA protein from *B. subtilis *forms oligomers that assemble into a highly ordered two-dimensional network, which is proposed to create the cell polarity needed for sporulation or tip extension [15]. More recently, *in vivo *and *in vitro *cross-linking experiments showed that Wag31 also forms homo-oligomers in *M. bovis *BCG [12]. Because our previous and current findings suggest that the phosphorylation of Wag31 play a regulatory role in polar peptidoglycan biosynthesis [3,11], we hypothesized that the phosphorylation state of Wag31 may affect its oligomerization at the cell poles by modulating interactions between Wag31 molecules, which in turn influence the peptidoglycan biosynthesis at the polar location.

**Figure 2:**
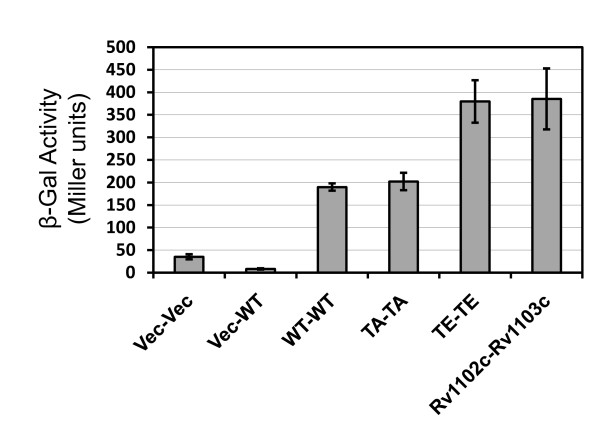
**Protein-protein interaction of Wag31 molecules by the yeast two-hybrid system**. The pJZ4-G and pHZ5-NRT clones with each *wag31_Mtb _*allele were individually transformed into the RFY231 and the Y309 strains, respectively. Four independent colonies from each transformation were mated, and reporter phenotypes for protein-protein interaction were determined by quantitative measurements of β-galactosidase activity using the Yeast β-Galactosidase Assay Kit (Pierce). WT-WT, interaction between Wag31_Mtb_-Wag31_Mtb_; TA-TA, interaction between Wag31T73A_Mtb_-Wag31T73A_Mtb_; TE-TE, interaction between Wag31T73E_Mtb_-Wag31T73E_Mtb_; Vec-WT, control containing pHZ5-NRT-*wag31_Mtb _*and pJZ4-G vector; Vec-Vec, control containing pHZ5-NRT and pJZ4-G vectors; Rv1102c-Rv1103c, positive control containing pHZ5-NRT-Rv1102c and pJZ4-G-Rv1103c [39]. Data shown are from a representative experiment done in duplicate, and data are represented as mean +/- SEM.

To address this hypothesis, we first determined whether the phosphorylation of Wag31 affects the protein-protein interaction between Wag31 molecules using the yeast two-hybrid system [16]. Wild-type Wag31_Mtb _showed interaction with itself, compatible with the finding of the Wag31 oligomerization in *M. bovis *BCG by Nguyen *et al*. (2007) (Figure 2). Interestingly, interaction between phosphomimetic Wag31T73E_Mtb _molecules was approximately two-fold stronger than the one between wild-type Wag31_Mtb _molecules or between phosphoablative Wag31T73A_Mtb _molecules, supporting a role of the phosphorylation in stimulating the interaction between Wag31 molecules. Interaction between wild-type Wag31 molecules was similar to that of Wag31T73A molecules, which is likely the result from lack of phosphorylation of wild-type Wag31 in the absence of the cognate Pkn's in *Saccharomyces cerevisiae*.

**Figure 3:**
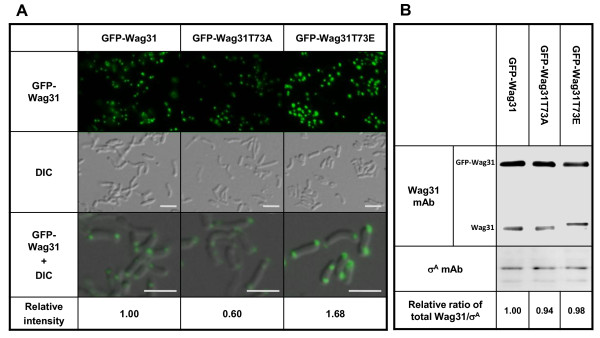
**Effect of Wag31 phosphorylation on polar localization**. A. Each *gfp-wag31_Mtb _*allele behind the P_acet _promoter in the replicating plasmid pMV261 (pCK174, pCK175 and pKC176) was introduced into the *wag31_Msm _*deletion mutant expressing *wag31_Mtb _*(KMS41), *wag31T73A_Mtb _*(KMS42) or *wag31T73E*_*Mtb *_(KMS43) under a P_tet _promoter, respectively. Transformants (KMS69, KMS70, and KMS71) were cultured in the presence of tetracycline (20 ng ml^-1^) until early-log phase where the expression of each *gfp*-*wag31 *allele was induced with acetamide (0.1%) for 3 hr before cells were observed under a fluorescence microscope, and the polar GFP-Wag31 signal was measured by using ImageJ software. Top, GFP signal from fluorescence microscopy; Middle, DIC image of the cells shown at the top panel; Bottom, enlarged overlap image of GFP signal and DIC. Average GFP intensity from cells expressing *gfp-wag31T73A_Mtb _*or *gfp- wag31T73E_Mtb _*relative to those of cells expressing wild-type *gfp-wag31 *is shown at the bottom. *p*-values for the difference in GFP signals (one-tailed, unpaired t-tests): wild-type Wag31_Mtb _vs. Wag31T73E_Mtb _= 1.2 × 10^-14^, significant, and wild-type Wag31_Mtb _vs. Wag31T73A_Mtb _= 1.2 × 10^-36^, significant (significant to p < 0.05). bar, 5 μm. B. Western blot analysis to examine the total Wag31 levels (GFP-Wag31 from P_acet _and non-tagged Wag31 from P_tet_) relative to those of SigA_Msm_. Total protein was purified from each strain at the same time cells were examine for fluorescence, and 20 μg of total protein was used for Western blot analysis with the anti-Wag31 mAb, stripped of the antibody, and subsequently for another Western blot with a monoclonal antibody against the Sig70 of *E. coli *RNA polymerase (Abcam). The ratio of total Wag31/SigA signal intensity from cells expressing wild-type *gfp-wag31 *was set as 1. Data shown are from a representative experiment done in duplicate.

Based on the yeast two-hybrid result, we predicted that the stronger interaction between the phosphorylated Wag31 molecules would lead to the enhanced localization of Wag31 to the polar regions. This prediction was tested by comparing the localization of GFP fused to Wag31_Mtb_, Wag31T73A_Mtb_, or Wag31T73E_Mtb _in the deletion mutants of *wag31_Msm _*expressing the corresponding *wag31 *allele (strains KMS69, KMS70, and KMS71). Quantification of polar GFP signals revealed that cells with Wag31T73E_Mtb _have 2.8-fold higher, and cells with wild-type Wag31_Mtb _have 1.7-fold higher GFP signals than cells with Wag31T73A_Mtb _(Figure 3A), while this increase in polar localization of wild-type Wag31 and Wag31T73E could be, in part, due to altered association of Wag31 with other unknown molecules. This difference in polar Wag31-GFP signals was not due to difference in the expression levels of Wag31_Mtb _because approximately equal levels of Wag31_Mtb _(sum of GFP-fused Wag31_Mtb _and non-tagged Wag31_Mtb_) relative to the levels of housekeeping SigA_Msm _were found from these stains (Figure 3B). In addition, such localization was not seen when GFP alone was expressed, indicating that the GFP-Wag31 localizations are not a GFP artifact (Additional file 2 (Fig. A1)).

**Figure 4:**
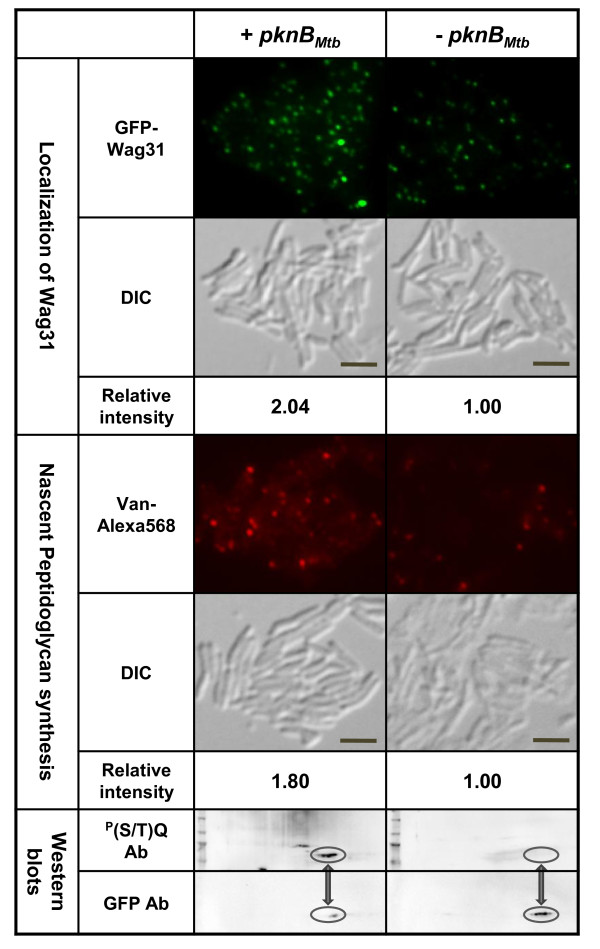
**Localization of Wag31 and nascent peptidoglycan biosynthesis in the presence or absence of *pknB_Mtb_*-overexpression**. Early-log phase cells of *M. smegmatis *(KMS4) containing pCK314 were divided into two flasks, and *pknB_Mtb _*was expressed in one of the flasks for 2 hr by adding 0.1% of acetamide. Subsequently, both cultures were incubated with tetracycline (20 ng ml^-1^) for 2 hr to express *gfp-wag31_Mtb _*and examined by a florescent microscope. For vancomycin staining, Van-Alexa568 (5 μg/ml) was added to each culture at the end of tetracycline induction and further incubated for 20 min before examining by a fluorescence microscope. Average GFP and Van-Alex568 intensity from cells with *pknB_Mtb_*-overexpression relative to that of cells without *pknB_Mtb_*-overexpression are shown at the bottom of each panel. (*p*-value for the difference in GFP signals = 1.63 × 10^-11^, and for Van-Alexa568 signals = 1.82 × 10^-7^). Phosphorylation of GFP-Wag31 by *pknB_Mtb_*-overexpression is shown at the bottom panel. 200 μg of total protein was used for 2-D PAGE and Western blot analysis with a phospho-(S/T)Q antibody, which was then stripped before conducting a subsequent Western blot with a GFP antibody. bar, 5 μm.

To further confirm the effect of the Wag31 phosphorylation on its polar localization, we examined the localization of wild-type Wag31_Mtb _in the presence or absence of *pknA_Mtb_*- or *pknB_Mtb_*-overexpression. We previously showed that Wag31 was weakly phosphorylated by PknA_Mtb_, which was significantly enhanced by the addition of PknB_Mtb _*in vitro *[3]. Consistent with this, *pknA*-overexpression only slightly increased the polar localization of Wag31 and polar peptidoglycan biosynthesis (Additional file 3 (Fig. A2)). However, overexpression of *pknB_Mtb_*, which dramatically increased the phosphorylation of GFP-Wag31 (Figure 4 bottom panel), elevated the polar localization of Wag31 (two-fold, upper panel) and nascent peptidoglycan biosynthesis (1.8-fold, middle panel) compared to cells without *pknB_Mtb_-*overexpression. These data further support that the phosphorylation of Wag31 enhances its polar localization, which in turn heightens polar peptidoglycan biosynthesis.

### Phosphorylation of Wag31 affects the enzymatic activity of the peptidoglycan biosynthetic pathway

Bacterial peptidoglycan synthesis is a complex process involving many different cytoplasmic and membrane steps [17]. In *Escherichia coli*, the cytoplasmic steps culminate in the formation of the UDP-MurNAc-(pentapeptide) catalyzed by a series of enzymatic activities of Mur proteins (MurA, MurB, MurC, MurD, MurE and MurF). The membrane-associated steps are then initiated with the formation of MurNAc-(pentapeptide)-diphosphoryl-undecaprenol (lipid I), a reaction catalyzed by MraY [18]. In a subsequent step by MurG, one GlcNAc residue is added to lipid I to form GlcNAc-MurNAc-(pentapeptide)-diphosphoryl-undecaprenol (lipid II), which is flipped to the outer surface of the membrane to be incorporated into the preexisting peptidoglycan by penicillin binding proteins. The structure of mycobacterial peptidoglycan is believed to be similar to that of *E. coli*, although it has a few differences [19]. The same appears to be true for its biosynthesis because *M. tuberculosis *possesses all eight *mur *genes that are present in *E. coli *[20].

**Figure 5:**
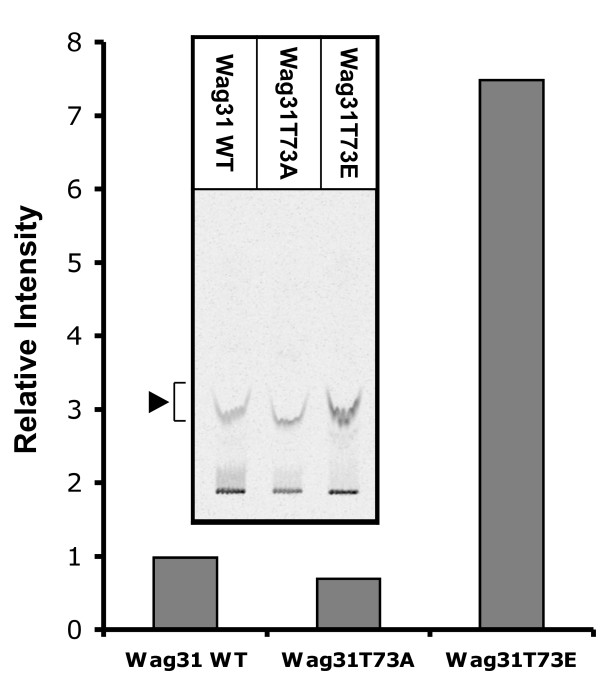
**Effect of Wag31 phosphorylation on enzymatic activity of MraY and MurG**. The three strains used in Fig. 1 were cultured to mid-log phase to purify a cell wall enriched envelope fraction (P60), which was then used as the sources of lipid (polyprenyl phosphate) and enzymes (MraY and MurG). 2 mg of P60 protein from each strain was incubated with 50 μM UDP-MurNAc-pentapeptide and 100 μM ATP for 5 min at 28°C, and reactions were initiated by adding 1 μCi of UDP-[^14^C]GlcNAc. After 1 hr, the quantity of radiolabeled lipid II (▶) was determined on TLC plates (inset) by a Phosphoimager. Data shown are from a representative experiment done in duplicate.

Our results described so far suggest that the phosphorylation of Wag31 has an influence on cell growth, at least in part, by regulating its polar localization and possibly the biosynthesis of peptidoglycan precursors. These data led us to hypothesize that Wag31 phosphorylation regulates polar peptidoglycan synthesis by affecting, directly or indirectly, the peptidoglycan synthetic machinery. To address this, the activity of Mur enzymes was determined among the *wag31_Msm _*deletion mutant strains expressing different *wag31 *alleles. We began with measuring the combined activity of MraY and MurG because these enzymes produce the final membrane-bound disaccharide-pentapeptide product. Briefly, a cell envelope-enriched fraction (P60) was purified from the *wag31 *deletion mutant cells expressing *wag31_Mtb_*, *wag31T73A_Mtb_*, or *wag31T73E_Mtb _*(strains KMS 41, KMS42, and KMS43), incubated with UDP-MurNAc-pentapeptide (substrate of MraY), ATP and UDP-[^14^C]GlcNAc, and then the reaction product of MurG ([^14^C]GlcNAc-MurNAc-(pentapeptide)-diphosphoryl-undecaprenol, lipid II) was analyzed by TLC [21,22]. Figure 5 shows that the P60 fraction from cells expressing *wag31T73E_Mtb _*produced 7.5-fold more ^14^C-labeled lipid II, but cells with *wag31T73A_Mtb _*yielded 30% less of this product than cells expressing wild-type *wag31_Mtb_*. In a repeated experiment with independently purified P60 fractions, a similar result was observed (the ratio of reaction product from cells with wild-type *wag31_Mtb _*: *wag31T73A_Mtb _*: *wag31T73E_Mtb _*were 1 : 0.65 : 5.3) (data not shown). These data suggested that the Wag31 phosphorylation, directly or indirectly, regulates the combined activity of MraY and MurG.

Consistent with this finding, we recently found in a Raman spectroscopic analyses that cells containing *wag31T73E_Mtb _*allele had increased intensity of the Raman peaks that have previously been attributed to D-glutamic acid, D-alanine, and *N*-acetylglucosamine components of peptidoglycan [23,24] than cells expressing wild-type *wag31_Mtb_*, which in turn showed higher intensity of the these peaks than cells with *wag31T73A_Mtb _*[25]. An increase in the intensity of these peaks suggests an increase in the quantity of these molecules, thus peptidoglycan in the cell. A corresponding pattern in the intensity of these peaks was also seen in the Raman spectra from the P60 cell envelope-enriched fractions, indicating that the increase of these molecules is localized in the membrane.

## Discussion

Our current results provide insights into a novel mechanism for the regulation of polar peptidoglycan synthesis in mycobacteria via differential polar localization of Wag31 depending on its phosphorylation status. This mechanism of the signal transduction system involving the Wag31 phosphorylation may be widespread among Gram-positive bacteria containing DivIVA because recent studies demonstrated that DivIVA in *Streptococcus agalactiae *and *Streptococcus pneumoniae *is also phosphorylated even though its function is yet to be discovered [26,27]. Since some bacteria such as *Mycobacterium *and *Corynebacterium *species lack MreB [2,9] but have a rod-like shape, and insert peptidoglycan at the cell poles instead of the helical pattern that uses actin-like MreB homologues, our data also suggest that Wag31 could serve as a determinant that directs peptidoglycan synthesis to the poles in mycobacterial cells. Interestingly, a recent study in *C. glutamicum *found that PknA_Cglu _phosphorylates, and thereby regulates, the activity of MurC [28]. In addition, in *M. tuberculosis*, GlmU, which catalyzes the formation of UDP-GlcNAc (the substrate of MurA), is phosphorylated by PknA_Mtb _and PknB_Mtb _*in vitro *[29], and another enzyme, MurD, is phosphorylated by PknA_Mtb _[30]. These findings suggest that PknA_Mtb _and PknB_Mtb _kinases are key regulatory components that modulate peptidoglycan biosynthesis and cell growth in mycobacteria via many targets including Wag31 and Mur enzymes.

What is the molecular mechanism by which Wag31 and its phosphorylation regulate the activity of peptidoglycan synthetic enzymes? Protein sequence alignments of Wag31 with DivIVA homologs revealed two conserved coiled-coil regions at the N- and C-termini, which are interrupted by a highly variable sequence, which includes the phosphorylation site of Wag31 [4]. Coiled-coil domains are known to function in protein-protein interactions [31], and the two coiled-coil regions in Wag31 may be responsible for the formation of oligomers of Wag31 *in vitro *and the potential superstructure *in vivo *as proposed [12,15]. These facts, taken together with our current finding of the phosphorylation-dependent localization of Wag31 thus tempted us to propose that the recruitment of Wag31 to the cell poles, which is mediated by interactions between coiled-coil regions of Wag31 molecules and is enhanced by the phosphorylation, modulates, directly or indirectly, the activity of peptidoglycan synthetic enzymes such as MraY and MurG. It is not clear, however, whether Wag31 affects these enzymes through direct interactions since we failed to detect the interactions between Mur enzymes and Wag31 (wild-type and phospho-mutants) in the yeast two-hybrid or mycobacterial protein fragment complementation system [32]. In addition, we were not able to reconstitute an assay system to test the effect of the Wag31 phosphorylation on the enzymatic activity of MraY and MurG *in vitro *because we could not purify these enzymes in *E. coli*, due to the toxicity of these enzymes when overexpressed. These negative results, however, suggested that the localization, and thus the activity, of Wag31 *in vivo *in *M. tuberculosis *is probably under tight regulation that involves multiple players.

In our previous studies, we showed that Wag31 is mainly phosphorylated during exponential phase where transcription of the *pknA/B_Mtb _*operon is high, and remains non- or lowly-phosphorylated during stationary phase as transcription of the *pknA*/*B_Mtb _*operon drops [3,11]. Thus, our current data support the following model. When mycobacterial cells are growing rapidly as in exponential phase, Wag31 is phosphorylated by the PknA/B_Mtb _kinases and strongly recruited to the cell poles to facilitate peptidoglycan biosynthesis so that enough peptidoglycan is produced to meet the demands of fast growth. It is possible that PknB_Mtb _senses this favorable external condition by detecting muropeptides through its PASTA repeats and regulates peptidoglycan biosynthesis through Wag31, because, in *B. subtilis*, the PrkC kinase, a homolog of PknB_Mtb _with three PASTA domains, induces germination in response to muropeptide fragments released by surrounding growing bacteria [33]. In stationary phase, however, Wag31 remains non- or lowly-phosphorylated but can still be recruited to the cell poles and lead to polar peptidoglycan synthesis. This idea is consistent with our observation that the phosphoablative Wag31T73A does localize at the cell poles (Figure 3A), and that wild-type GFP-Wag31 shows clear localization and peptidoglycan biosynthesis at cell poles at late stationary phase, albeit lower than in exponential phase (data not shown). This model is also consistent with previous reports that a fairly high capacity for peptidoglycan biosynthesis is maintained in slow-growing and stationary phase bacterial cells [34]. Either way, Wag31 itself is essential for mycobacterial survival as we observed in our previous report [11] because Wag31 must be present and localized to the cell poles for polar peptidoglycan synthesis.

## Conclusions

This study demonstrated that Wag31_Mtb _phosphorylation, which is unique among DivIVA homologues, regulates polar peptidoglycan biosynthesis and optimal growth of mycobacterial cells through modulating the localization of Wag31 and the activity of peptidoglycan biosynthetic enzymes.

## Methods

### Bacterial growth condition, media and strains

*M. smegmatis *mc^2^155 cultures were grown at 37°C in Middlebrook 7H9 liquid medium (Difco) supplemented with 10% ADC (5% (W/V) BSA fraction V, 2% (W/V) glucose and 0.85% (W/V) NaCl) and 0.05% (W/V) Tween-80, or on Middlebrook 7H9-ADC agar plates. Kanamycin (50 μg ml^-1^), hygromycin (50 μg ml^-1^) or apramycin (50 μg ml^-1^) was added to culture media as indicated. *E. coli *TOP10 strain (Life Technologies) was used as host strain for cloning experiments, and was grown in LB broth or solid medium with kanamycin (20 μg ml^-1^).

### Plasmid construction

All plasmid constructs and primers are shown in Additional file 1 and 4 (Table A1 and A2). For localization of different forms of Wag31_Mtb_, wild-type *gfp-wag31_Mtb_*, *gfp-wag31T73A_Mtb _*or *gfp-wag31T73E_Mtb _*was cloned under the acetamide-inducible promoter (P_acet_) in a replicating plasmid pMV261 (Km^r^) to make pCK174, pCK175, and pCK176, respectively. The *gfp *gene was amplified from pTracerCMV plasmid (Invitrogen) using Ngfp-wag-1 and Ngfp-wag-2 primers. Genes for Wag31_Mtb_, Wag31T73A_Mtb _and Wag31T73E_Mtb _were amplified from plasmids pCK89, pCK90, and pCK91 using Ngfp-TBwag-3 and Ngfp-TBwag-4 primers. Second overlap PCR to fuse *gfp *and each *wag31_Mtb _*gene was conducted by using Ngfp-wag-1 and Ngfp-TBwag-4 primers. To test localization of Wag31 in the presence of *pknA_Mtb_*- or *pknB_Mtb_*-overexpression in *M. smegmatis*, wild-type *gfp*-*wag31_Mtb _*was cloned under tetracycline-inducible promoter (P_tet_) in a replicating plasmid pMV261 (Hyg^r^) to make pCK314 by using primers GFPWag-Bam-5 and GFPWag-Cla-Spe-3 [11]. For the yeast two-hybrid study, each *wag31_Mtb _*allele was cloned in frame into both pJZ4-G (pCK145, pCK143, and pCK142) and pHZ5-NRT vectors (pCK146, pCK147, and pCK148) [35]. Each *wag31 *allele was amplified by PCR using the WagYTHF and WagYTHR primers, and pCK89, pCK90, and pCK91 as the templates.

### Nascent peptidoglycan biosynthesis and localization of Wag31

For observation of nascent peptidoglycan biosynthesis, the *wag31_Msm _*deletion mutant cells of *M. smegmatis *containing P_tet_-*wag31_Mtb _*(pCK89), P_tet_-*wag31T73A_Mtb _*(pCK90), or P_tet_-*wag31T73E_Mtb _*(pCK91) or cells containing pMV261-P_tet_-*wag31 *(pCK314) with or without *pknA_Mtb_*- (KMS 2) or *pknB_Mtb_*-overexpression (KMS 4) were stained with Van-Alexa568 [11]. A stock solution of Van-alexa568 (5 mg ml^-1^) was prepared according to the manufacturer's manual (Molecular Probes). Each strain was cultured in 7H9 liquid medium with tetracycline (20 ng ml^-1^) overnight and was then inoculated into fresh 7H9 liquid medium containing 20 ng ml^-1 ^of tetracycline. Cells from each strain were taken during mid-log phase (approximate OD_600 _= 0.4) and incubated with Van-alexa568 (5 μg ml^-1^) for 20 min at 37°C. For microscopic analysis, cells were washed with PBS buffer and examined by an Olympus BX51 microscope. Pictures were taken with an Olympus DP30BW high sensitivity cooled CCD camera, acquired with DP-BSW software and processed with Adobe Photoshop CS2. To minimize possible errors during the sampling process and fluorescence examination, the staining procedure was conducted in the dark, and microscopy conditions such as exposure time and opening of the aperture diaphragm were fixed for all samples. For quantification of average fluorescence intensity at the cell poles, DIC and fluorescence images were superimposed to align cells and fluorescence signals, and fluorescence density from the poles of approximately 300 cells was measured and background-corrected by using the ImageJ software.

For localization of different forms of Wag31, pMV261 containing P_acet_-*gfp*-*wag31_Mtb _*(pCK174), P_acet_-*gfp-wag31T73A_Mtb _*(pCK175) or P_acet_-*gfp-wag31T73E_Mtb _*(pCK176) was electroporated into the *wag31_Msm _*deletion mutant expressing *wag31_Mtb _*(KMS41), *wag31T73A_Mtb _*(KMS42) or *wag31T73E*_*Mtb *_(KMS43) under a tetracycline-inducible P_tet _promoter [36] at the chromosomal L5 *attB *locus, respectively. The resulting strains (KMS69, KMS70, and KMS71) were grown in 7H9 liquid medium containing 20 ng tetracycline, and at early-log phase (approximate OD_600 _= 0.2) cells were induced with 0.1% of acetamide for 3 hr before being transferred onto a glass slide and observed using an Olympus BX51 florescence microscope. Quantification of GFP signals at the cell poles of approximately 300 cells was conducted with ImageJ software similar to the one for Van-Alexa568. Examination of the Wag31 (both GFP-Wag31 and non-tagged Wag31) and SigA_Msm _protein levels at the end of acetamide induction was conducted using 20 μg of total protein and Western blots with the anti-Wag31 mAb [37] and a monoclonal antibody against the Sig70 subunit of *E. coli *RNA polymerase (Abcam), which also recognizes SigA of *M. smegmatis *[38]. Western signals were quantified by using the Quantity One software (Bio-Rad). To test the localization of wild-type Wag31 in the presence or absence of *pknA_Mtb_*- or *pknB_Mtb_*-overexpression, pCK314 was transformed into a *M. smegmatis *strain KMS2 or KMS4. Transformants were grown in 7H9 liquid medium until early-log phase (approximate OD_600 _= 0.2), split into two flasks, and 0.1% acetamide was added to express *pknA_Mtb _*or *pknB_Mtb _*for 2 hr. Both cultures were further incubated with tetracycline (20 ng ml^-1^) for 2 hr to express *gfp-wag31_Mtb_*. For Van-Alexa568 staining, 5 μg ml^-1 ^of Van-Alexa568 was added to both cultures, and incubated for 20 min at 37°C before microscopic examination. To examine the phosphorylation of wild-type Wag31_Mtb _under *pknB_Mtb_*-overexpression, total protein was purified and cleaned up with the ReadyPrep 2 D Cleanup Kit (Bio-Rad). 200 μg of total protein from each sample was rehydrated into isoelectric focusing strips with a pH range of 4 to 7 (Bio-Rad). Isoelectric focusing was performed for 35,000 V-h in a PROTEAN IEF Cell (Bio-Rad). 2-D SDS-PAGE was performed using 10% Tris-HCl gels (Bio-Rad), and immunoblot blot analysis was performed using a phospho-(S/T)Q polyclonal antibody (Cell Signaling Technology), stripped, and then re-probed with anti-GFP antibody (Sigma).

### Yeast two-hybrid analysis

Constructs of pJZ4-G-*wag31 *(pCK145), pJZ4-G-*wag31T73A *(pCK143) and pJZ4-G-*wag31T73E *(pCK142) were individually transformed into the yeast strain RFY231 by plating on agar minimal media lacking tryptophan [16]. Each of pHZ5-NRT-*wag31 *(pCK146), pHZ5-NRT-*wag31T73A *(pCK147) and pHZ5-NRT-*wag31T73E *(pCK148) was also transformed into another yeast strain Y309 by plating on agar minimal media lacking histidine and uracil. Four independent colonies from each transformation were mated on YPD plates, re-streaked onto minimal media lacking uracil, histidine, and tryptophan. As negative controls, mated cells containing empty vectors alone or cells containing pHZ5-NRT-*wag31_Mtb _*(pCK146) and pJZ4-G vector were included. Mated cells that we recently showed the interaction between Rv1102c and Rv1103c (with pCK227 and pCK228) [39] were included as a positive control. Quantitative measurements (β-galactosidase activity in Miller unit) of interactions were conducted by using the Yeast β-Galactosidase Assay Kit (Pierce).

### Enzymatic assay for peptidoglycan synthetic enzymes

The *wag31_Msm _*deletion mutants containing each *wag31_Mtb _*allele behind the P_tet _promoter (KMS41, KMS42, and KMS43) were cultured to mid-log phase (approximate OD_600 _= 0.4), and a cell-wall enriched envelope fraction (P60) was prepared as previously described [22]. Briefly, 8 g of harvested cells were resuspended in 30 ml of buffer A (50 mM MOPS (pH 8.0), 10 mM MgCl_2_, and 5 mM β-mercaptoethanol), and subjected to probe sonication using 10 cycles of 60 sec with 90 sec cooling on ice between the cycles. After centrifugation at 23,000 × g for 30 min at 4°C, the pellet was resuspended in buffer A with 60% Percoll (GE Healthcare), followed by centrifugation at 23,000 × g for 60 min at 4°C. The upper, flocculent band was recovered and washed with buffer A three times, to remove residual Percoll. The cell wall enriched pellet containing cell wall and some residual membrane was then resuspended in buffer A using a Dounce homogenizer. These P60 fractions were used as the sources of lipid (polyprenyl phosphate) and enzymes (MraY and MurG).

For enzymatic assay, reaction mixtures containing 2 mg of P60 protein from each strain, 50 μM UDP-MurNAc-pentapeptide and 100 μM ATP in a reaction volume of 300 μl with buffer A were incubated for 5 min at 28°C. Reactions were initiated by adding 1 μCi of UDP-[^14^C]GlcNAc (Perkin Elmer Life Sciences) and incubated at 28°C. After 1 hr, reactions were terminated by addition of 20 volumes of CHCl_3_/CH_3_OH (2:1), centrifuged at 3,000 × g for 10 min at room temperature, and the supernatant was mixed with 0.6 ml of dH_2_O in a new tube. The resulting biphasic solution was centrifuged again and the upper, aqueous phase was discarded. The bottom, organic phase was washed with 1.5 ml of CHCl_3_/CH_3_OH/H_2_O (3:47:48), dried under a stream of N_2 _and re-dissolved in CHCl_3_/CH_3_OH/H_2_O/NH_4_OH (65:25:3.6:0.5). The recovered radioactive materials were applied to a silica gel TLC plate, which was developed with CHCl_3_/CH_3_OH/H_2_O/NH_4_OH (5.6:4.2:0.68:0.27). The location and quantity of radiolabeled lipid II ([^14^C]GlcNAc-MurNAc-(pentapeptide)-diphosphoryl-undecaprenol) on the TLC plate was determined by using a Molecular Dynamics Typhoon 8600 Phosphoimager (Molecular Dynamics).

## Abbreviations

Mtb: *Mycobacterium tuberculosis*; Msm: *Mycobacterium smegmatis*; MurNAc-(pentapeptide): *N*-acetylmuramyl-(pentapeptide); GlcNAc: *N*-acetylglucosamine; DIC: Differential interference contrast

## Authors' contributions

CJ, HE, and JJL participated in the design and conduct of the study, data analysis, and drafting of manuscript. In particular, CJ conducted Van-Alexa568 staining and localization of Wag31 in cells expressing different mutant form of Wag31. HE conducted the enzymatic assay of Mur proteins. JJL performed the yeast two-hybrid experiments and Van-Alexa568 and localization of wild-type Wag31 in the presence of kinase overexpression. KH and MBS participated in culture and isolation of P60 samples for Mur enzyme assays and Raman spectrometry. JSH, SN and JYL constructed plasmids for localization and yeast two-hybrid assay. JWS, SHL, and SJR participated in the data analysis, and drafting and revision of the manuscript. DCC participated in the conception and design of the study, general supervision of the Mur enzyme assays. CMK participated in the design of the study, general supervision of the research, and critical revision of the manuscript. All authors read and approved the final version of the manuscript.

## Supplementary Material

Additional file 1 **Table A1: List of strains and plasmids used in this study**. List of plasmid constructs and strains made for this study. Click here for file

Additional file 2 **Fig. A1: Control *M. smegmatis *expressing *gfp *alone**. A control experiment in *M. smegmatis *to show that GFP-Wag31 localization is not due to the non-specific localization of GFP itself. Click here for file

Additional file 3 **Fig. A2: Localization of Wag31 and nascent peptidoglycan biosynthesis in the presence or absence of *pknA_Mtb_*-overexpression**. Examination of wild-type Wag31 localization and polar peptidoglycan biosynthesis when *pknA *is overexpressed in *M. smegmatis*. Click here for file

Additional file 4 **Table A2: Primers used in this study**. List of primers used to make plasmid constructs for this study. Click here for file

## References

[B1] WHO Tuberculosis Facts Sheet 2007

[B2] Cole ST Brosch R Parkhill J Garnier T Churcher C Harris D Gordon SV Eiglmeier K Gas S Barry CE Deciphering the biology of *Mycobacterium tuberculosis *from the complete genome sequence Nature 1998 393 537 544 10.1038/31159 9634230

[B3] Kang CM Abbott DW Park ST Dascher CC Cantley LC Husson RN The *Mycobacterium tuberculosis *serine/threonine kinases PknA and PknB: substrate identification and regulation of cell shape Genes & Development 2005 19 1692 1704 10.1101/gad.1311105 PMC1176007 15985609

[B4] Flardh K Essential role of DivIVA in polar growth and morphogenesis in *Streptomyces coelicolor *A3(2) Mol Microbiol 2003 49 1523 1536 10.1046/j.1365-2958.2003.03660.x 12950918

[B5] Cha JH Stewart GC The divIVA minicell locus of *Bacillus subtilis* Journal of Bacteriology 1997 179 1671 1683 9045828 10.1128/jb.179.5.1671-1683.1997 PMC178881

[B6] Thomaides HB Freeman M El Karoui M Errington J Division site selection protein DivIVA of *Bacillus subtilis *has a second distinct function in chromosome segregation during sporulation Genes Dev 2001 15 1662 1673 10.1101/gad.197501 11445541 PMC312724

[B7] Marston AL Errington J Selection of the midcell division site in *Bacillus subtilis *through MinD-dependent polar localization and activation of MinC Molecular Microbiology 1999 33 84 96 10.1046/j.1365-2958.1999.01450.x 10411726

[B8] Marston AL Thomaides HB Edwards DH Sharpe ME Errington J Polar localization of the MinD protein of *Bacillus subtilis *and its role in selection of the mid-cell division site Genes & Development 1998 12 3419 3430 10.1101/gad.12.21.3419 PMC317235 9808628

[B9] Letek M Ordonez E Vaquera J Margolin W Flardh K Mateos LM Gil JA DivIVA is required for polar growth in the MreB-lacking rod-shaped actinomycete *Corynebacterium glutamicum* J Bacteriol 2008 190 3283 3292 10.1128/JB.01934-07 18296522 PMC2347398

[B10] Ramos A Honrubia MP Valbuena N Vaquera J Mateos LM Gil JA Involvement of DivIVA in the morphology of the rod-shaped actinomycete *Brevibacterium lactofermentum* Microbiology 2003 149 3531 3542 10.1099/mic.0.26653-0 14663085

[B11] Kang CM Nyayapathy S Lee JY Suh JW Husson RN Wag31, a homologue of the cell division protein DivIVA, regulates growth, morphology and polar cell wall synthesis in mycobacteria Microbiology 2008 154 725 735 10.1099/mic.0.2007/014076-0 18310019

[B12] Nguyen L Scherr N Gatfield J Walburger A Pieters J Thompson CJ Antigen 84, an Effector of Pleiomorphism in *Mycobacterium smegmatis* J Bacteriol 2007 189 7896 7910 10.1128/JB.00726-07 17766411 PMC2168712

[B13] Mukherjee P Sureka K Datta P Hossain T Barik S Das KP Kundu M Basu J Novel role of Wag31 in protection of mycobacteria under oxidative stress Mol Microbiol 2009 73 103 119 10.1111/j.1365-2958.2009.06750.x 19496931

[B14] Daniel RA Errington J Control of cell morphogenesis in bacteria: two distinct ways to make a rod-shaped cell Cell 2003 113 767 776 10.1016/S0092-8674(03)00421-5 12809607

[B15] Stahlberg H Kutejova E Muchova K Gregorini M Lustig A Muller SA Olivieri V Engel A Wilkinson AJ Barak I Oligomeric structure of the *Bacillus subtilis *cell division protein DivIVA determined by transmission electron microscopy Mol Microbiol 2004 52 1281 1290 10.1111/j.1365-2958.2004.04074.x 15165232

[B16] Kolonin MG Zhong J Finley RL Interaction mating methods in two-hybrid systems Methods Enzymol 2000 328 26 46 10.1016/S0076-6879(00)28388-2 11075336

[B17] van Heijenoort J Recent advances in the formation of the bacterial peptidoglycan monomer unit Nat Prod Rep 2001 18 503 519 10.1039/a804532a 11699883

[B18] van Heijenoort J Lipid intermediates in the biosynthesis of bacterial peptidoglycan Microbiol Mol Biol Rev 2007 71 620 635 10.1128/MMBR.00016-07 18063720 PMC2168651

[B19] Mahapatra S Yagi T Belisle JT Espinosa BJ Hill PJ McNeil MR Brennan PJ Crick DC Mycobacterial lipid II is composed of a complex mixture of modified muramyl and peptide moieties linked to decaprenyl phosphate J Bacteriol 2005 187 2747 2757 10.1128/JB.187.8.2747-2757.2005 15805521 PMC1070386

[B20] Crick DC Mahapatra S Brennan PJ Biosynthesis of the arabinogalactan-peptidoglycan complex of *Mycobacterium tuberculosis* Glycobiology 2001 11 107R 118R 10.1093/glycob/11.9.107R 11555614

[B21] Crick DC Schulbach MC Zink EE Macchia M Barontini S Besra GS Brennan PJ Polyprenyl phosphate biosynthesis in *Mycobacterium tuberculosis *and Mycobacterium smegmatis J Bacteriol 2000 182 5771 5778 10.1128/JB.182.20.5771-5778.2000 11004176 PMC94699

[B22] Khasnobis S Zhang J Angala SK Amin AG McNeil MR Crick DC Chatterjee D Characterization of a specific arabinosyltransferase activity involved in mycobacterial arabinan biosynthesis Chem Biol 2006 13 787 795 10.1016/j.chembiol.2006.05.016 16873027

[B23] Sengupta A Brar N Davis EJ Bioaerosol detection and characterization by surface-enhanced Raman spectroscopy J Colloid Interface Sci 2007 309 36 43 10.1016/j.jcis.2007.02.015 17362975

[B24] Laucks ML Sengupta A Junge K Davis EJ Swanson BD Comparison of psychro-active arctic marine bacteria and common mesophillic bacteria using surface-enhanced Raman spectroscopy Appl Spectrosc 2005 59 1222 1228 10.1366/000370205774430891 16274534

[B25] Hamasha K Sahana MB Jani C Nyayapathy S Kang CM Rehse SJ The effect of Wag31 phosphorylation on the cells and the cell envelope fraction of wild-type and conditional mutants of *Mycobacterium smegmatis *studied by visible-wavelength Raman spectroscopy Biochem Biophys Res Commun 2010 391 664 668 10.1016/j.bbrc.2009.11.117 19932688

[B26] Silvestroni A Jewell KA Lin WJ Connelly JE Ivancic MM Tao WA Rajagopal L Identification of serine/threonine kinase substrates in the human pathogen group B streptococcus J Proteome Res 2009 8 2563 2574 10.1021/pr900069n 19309132 PMC2863997

[B27] Novakova L Bezouskova S Pompach P Spidlova P Saskova L Weiser J Branny P Identification of multiple substrates of the StkP Ser/Thr protein kinase in *Streptococcus pneumoniae* J Bacteriol 2010 192 3629 3638 10.1128/JB.01564-09 20453092 PMC2897338

[B28] Fiuza M Canova MJ Patin D Letek M Zanella-Cleon I Becchi M Mateos LM Mengin-Lecreulx D Molle V Gil JA The MurC Ligase Essential for Peptidoglycan Biosynthesis Is Regulated by the Serine/Threonine Protein Kinase PknA in *Corynebacterium glutamicum* J Biol Chem 2008 283 36553 36563 10.1074/jbc.M807175200 18974047 PMC2662310

[B29] Parikh A Verma SK Khan S Prakash B Nandicoori VK PknB-mediated phosphorylation of a novel substrate, N-acetylglucosamine-1-phosphate uridyltransferase, modulates its acetyltransferase activity J Mol Biol 2009 386 451 464 10.1016/j.jmb.2008.12.031 19121323

[B30] Thakur M Chakraborti PK Ability of PknA, a mycobacterial eukaryotic-type serine/threonine kinase, to transphosphorylate MurD, a ligase involved in the process of peptidoglycan biosynthesis Biochem J 2008 415 27 33 10.1042/BJ20080234 18557704

[B31] Herrmann H Haner M Brettel M Ku NO Aebi U Characterization of distinct early assembly units of different intermediate filament proteins Journal of Molecular Biology 1999 286 1403 1420 10.1006/jmbi.1999.2528 10064706

[B32] Singh A Mai D Kumar A Steyn AJ Dissecting virulence pathways of *Mycobacterium tuberculosis *through protein-protein association Proceedings of the National Academy of Sciences of the United States of America 2006 103 11346 11351 10.1073/pnas.0602817103 16844784 PMC1544089

[B33] Shah IM Laaberki MH Popham DL Dworkin J A eukaryotic-like Ser/Thr kinase signals bacteria to exit dormancy in response to peptidoglycan fragments Cell 2008 135 486 496 10.1016/j.cell.2008.08.039 18984160 PMC2892110

[B34] Mengin-Lecreulx D van Heijenoort J Effect of growth conditions on peptidoglycan content and cytoplasmic steps of its biosynthesis in *Escherichia coli* J Bacteriol 1985 163 208 212 3891726 10.1128/jb.163.1.208-212.1985 PMC219099

[B35] Finley RL Jr Zhang H Zhong J Stanyon CA Regulated expression of proteins in yeast using the MAL61-62 promoter and a mating scheme to increase dynamic range Gene 2002 285 49 57 10.1016/S0378-1119(02)00420-1 12039031

[B36] Blokpoel MC Murphy HN O'Toole R Wiles S Runn ES Stewart GR Young DB Robertson BD Tetracycline-inducible gene regulation in mycobacteria Nucleic Acids Research 2005 33 e22 10.1093/nar/gni023 15687380 PMC548381

[B37] Hermans PW Abebe F Kuteyi VI Kolk AH Thole JE Harboe M Molecular and immunological characterization of the highly conserved antigen 84 from *Mycobacterium tuberculosis *and *Mycobacterium leprae* Infection & Immunity 1995 63 954 960 10.1128/iai.63.3.954-960.1995 PMC173095 7868268

[B38] Predich M Doukhan L Nair G Smith I Characterization of RNA polymerase and two sigma-factor genes from *Mycobacterium smegmatis* Mol Microbiol 1995 15 355 366 10.1111/j.1365-2958.1995.tb02249.x 7746156

[B39] Han J-S Lee JJ Anandan T Zeng M Sripathi S Jahng WJ Lee SS Suh JW Kang CM Characterization of a chromosomal toxin-antitoxin, Rv1102c-Rv1103c system in *Mycobacterium tuberculosis* Biochemical and Biophysical Research communications 2010 in press 10.1016/j.bbrc.2010.08.023 20705052

[B40] Snapper SB Melton RE Mustafa S Kieser T Jacobs WR Jr Isolation and characterization of efficient plasmid transformation mutants of *Mycobacterium smegmatis* Mol Microbiol 1990 4 1911 1919 10.1111/j.1365-2958.1990.tb02040.x 2082148

