# Supplementary material for: Regulation of Polar Peptidoglycan Biosynthesis by Wag31 Phosphorylation in Mycobacteria
Source: BMC Microbiol. 2010 Dec 29;10:327. doi: 10.1186/1471-2180-10-327 (PMC3019181; doi:10.1186/1471-2180-10-327)
Supplement: Additional file 1 — Table A1: List of strains and plasmids used in this study. List of plasmid constructs and strains made for this study. [file 1471-2180-10-327-S1.DOCX]

**Additional file 1 (Table A1). List of strains and plasmids used in this study**

**Strain or plasmid** **Description**  **Reference or source**

**Strains**

*E. coli*

Top10 *E. coli* host for molecular cloning Invitrogen

*S. cerevisiae*

RFY231 the prey yeast host [16]

(*MATα, his3, trp1Δ::hisG, ura3,*

*3LexAop-Leu2::leu2 MAL^+^*)

Y309 the bait yeast host [16]

(*MATa*, *trp1Δ::hisG, his3Δ200, leu2-3,*

*lys2Δ201, ura3-52, mal-,* pSH18-34 (URA3+))

*M. smegmatis*

mc^2^155 wild-type strain of *M. smegmatis* [40]

KMS2 *M. smegmatis* carrying P_acet_-*pknA_Mtb_* at *attB* locus [11]

KMS4 *M. smegmatis* carrying P_acet_-*pknB_Mtb_* at *attB* locus [11]

KMS41 *M. smegmatis* *wag31_Msm_* deletion mutant carrying [11]

P_tet_-*wag31_Mtb_* (pCK89) at *attB* locus

KMS42 *M. smegmatis* *wag31_Msm_* deletion mutant carrying [11]

P_tet_-*wag31T73A_Mtb_* (pCK89) at *attB* locus

KMS43 *M. smegmatis* *wag31_Msm_* deletion mutant carrying [11]

P_tet_-*wag31T73E_Mtb_* (pCK89) at *attB* locus

KMS46 KMS41 carrying pCK76 [11]

KMS69 KMS41 carrying pCK174 This study

KMS70 KMS42 carrying pCK175 This study

KMS71 KMS43 carrying pCK176 This study

**Plasmids**

pJZ4-G yeast two-hybrid vector with the B42 activating domain [16]

pHZ5-NRT yeast two-hybrid vector with the LexA DNA-binding [16]

domain

pCK76 pMV261 (Km^r^) carrying P_acet_-*gfp* This study

pCK89 pMH94 (Apra^r^) carrying P_tet_-*wag31_Mtb_* [11]

pCK90 pMH94 (Apra^r^) carrying P_tet_-*wag31T73A_Mtb_* [11]

pCK91 pMH94 (Apra^r^) carrying P_tet_-*wag31T73E_Mtb_* [11]

pCK142 pJZ4-G carrying *wag31T73E_Mtb_*  This study

pCK143 pJZ4-G carrying *wag31T73A_Mtb_* This study

pCK145 pJZ4-G carrying *wag31_Mtb_* This study

pCK146 pHZ5-NRT carrying *wag31_Mtb_*  This study

pCK147 pHZ5-NRT carrying *wag31T73A_Mtb_* This study

pCK148 pHZ5-NRT carrying *wag31T73E_Mtb_* This study

pCK174 pMV261 (Km^r^) carrying P_acet_-*gfp-wag31_Mtb_* This study

pCK175 pMV261 (Km^r^) carrying P_acet_-*gfp-wag31T73A_Mtb_* This study

pCK176 pMV261 (Km^r^) carrying P_acet_-*gfp-wag31T73E_Mtb_* This study

pCK227 pJZ4-G carrying the Rv1103c gene [39]

pCK228 pHZ5-NRT carrying the Rv1102c gene [39]

pCK314 pMV261 (Hyg^r^) carrying P_tet_-*gfp-wag31_Mtb_* This study
