# Supplementary material for: Regulation of Polar Peptidoglycan Biosynthesis by Wag31 Phosphorylation in Mycobacteria
Source: BMC Microbiol. 2010 Dec 29;10:327. doi: 10.1186/1471-2180-10-327 (PMC3019181; doi:10.1186/1471-2180-10-327)
Supplement: Additional file 2 — Fig. A1: Control M. smegmatis expressing gfp alone. A control experiment in M. smegmatis to show that GFP-Wag31 localization is not due to the non-specific localization of GFP itself. [file 1471-2180-10-327-S2.PPT]

## Slide 1
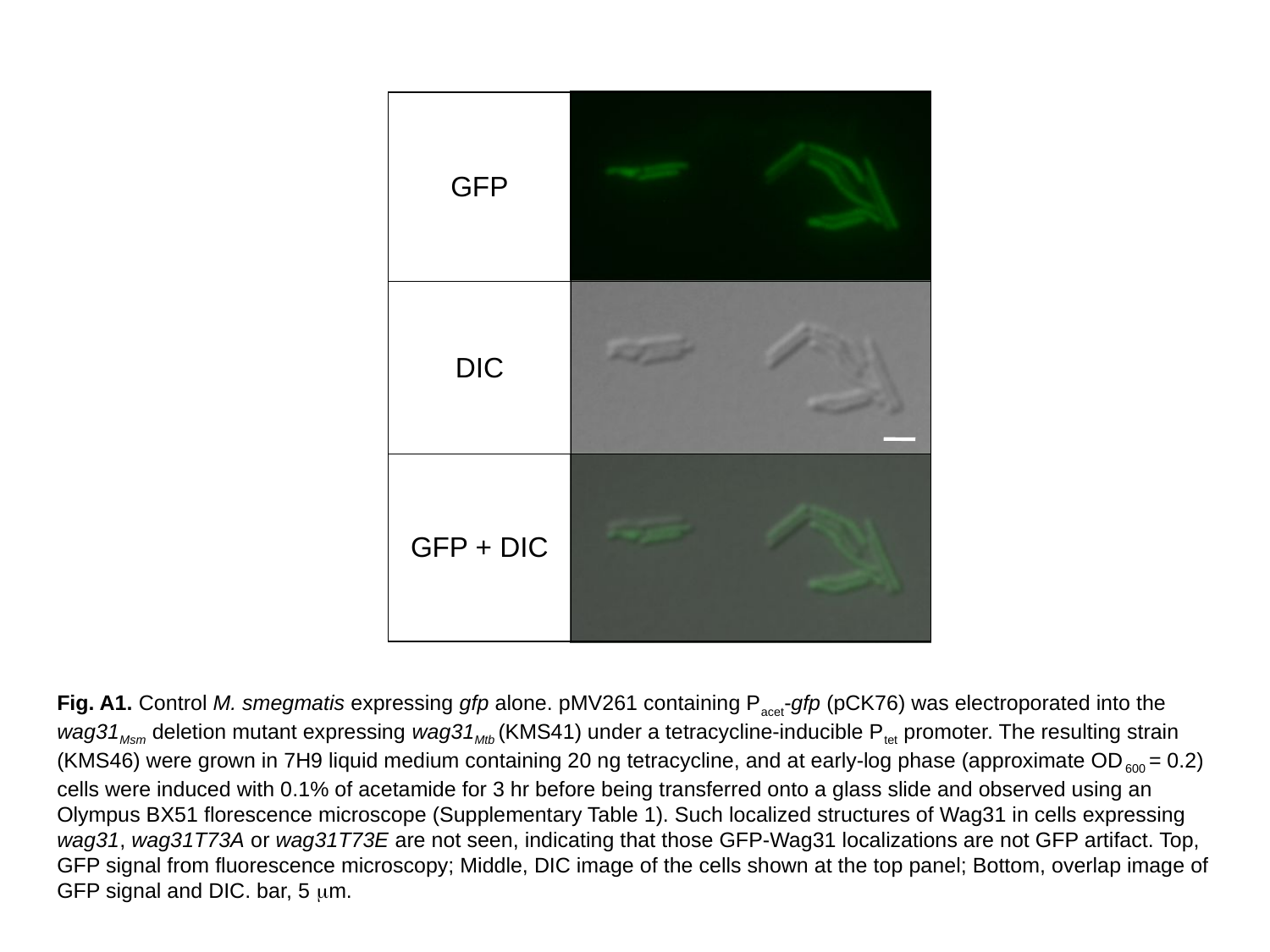

| GFP | |
| --- | --- |
| DIC | |
| GFP + DIC | |
Fig. A1. Control M. smegmatis expressing gfp alone. pMV261 containing Pacet-gfp (pCK76) was electroporated into the wag31Msm deletion mutant expressing wag31Mtb (KMS41) under a tetracycline-inducible Ptet promoter. The resulting strain (KMS46) were grown in 7H9 liquid medium containing 20 ng tetracycline, and at early-log phase (approximate OD600 = 0.2) cells were induced with 0.1% of acetamide for 3 hr before being transferred onto a glass slide and observed using an Olympus BX51 florescence microscope (Supplementary Table 1). Such localized structures of Wag31 in cells expressing wag31, wag31T73A or wag31T73E are not seen, indicating that those GFP-Wag31 localizations are not GFP artifact. Top, GFP signal from fluorescence microscopy; Middle, DIC image of the cells shown at the top panel; Bottom, overlap image of GFP signal and DIC. bar, 5 μm.
