# Supplementary material for: Regulation of Polar Peptidoglycan Biosynthesis by Wag31 Phosphorylation in Mycobacteria
Source: BMC Microbiol. 2010 Dec 29;10:327. doi: 10.1186/1471-2180-10-327 (PMC3019181; doi:10.1186/1471-2180-10-327)
Supplement: Additional file 3 — Fig. A2: Localization of Wag31 and nascent peptidoglycan biosynthesis in the presence or absence of pknAMtb-overexpression. Examination of wild-type Wag31 localization and polar peptidoglycan biosynthesis when pknA is overexpressed in M. smegmatis. [file 1471-2180-10-327-S3.PPT]

## Slide 1
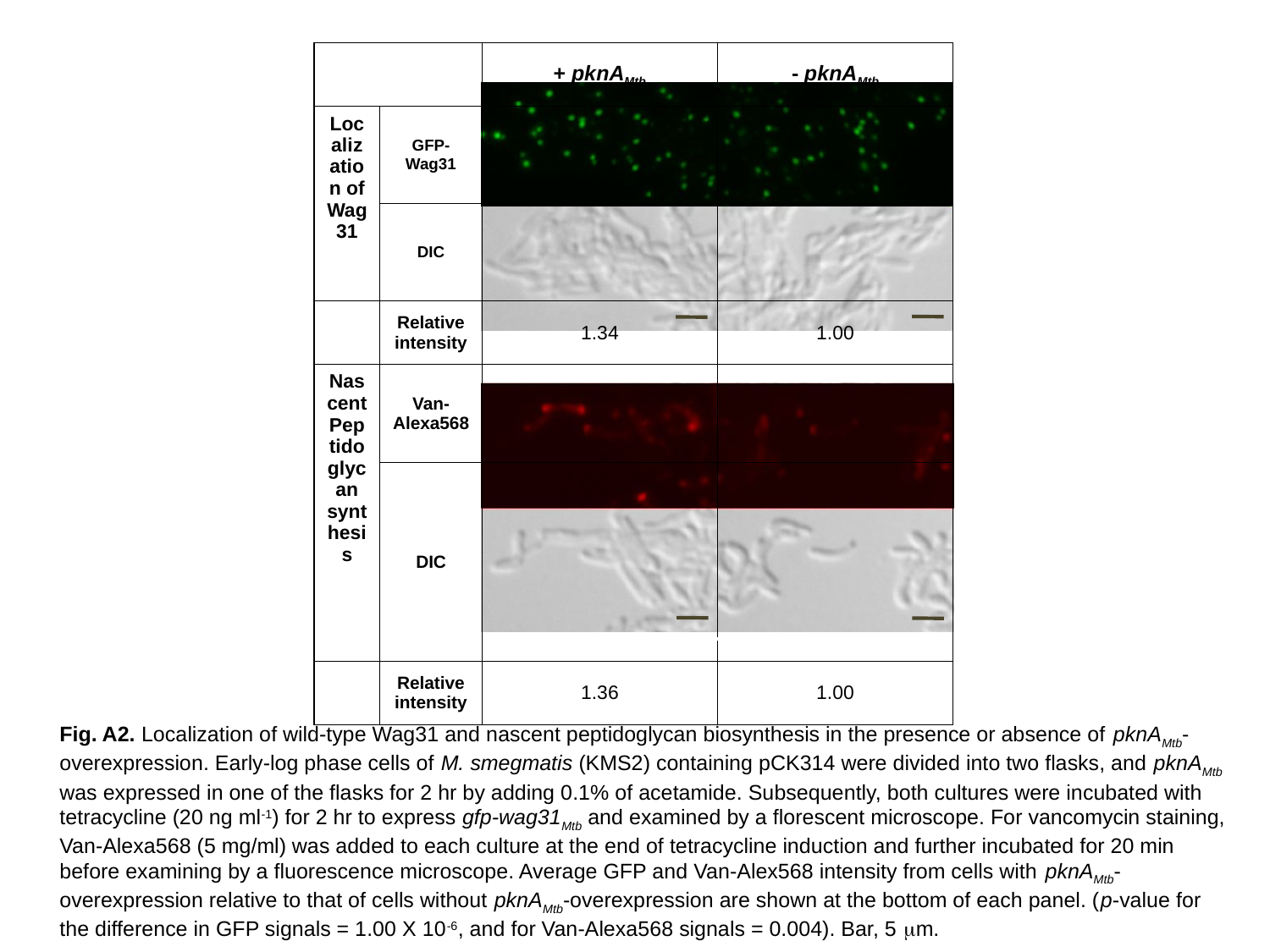

| | | + pknAMtb | - pknAMtb |
| --- | --- | --- | --- |
| Localization of Wag31 | GFP-Wag31 | | |
| | DIC | | |
| | Relative intensity | 1.34 | 1.00 |
| Nascent Peptidoglycan synthesis | Van-Alexa568 | | |
| | DIC | | |
| | Relative intensity | 1.36 | 1.00 |
Fig. A2. Localization of wild-type Wag31 and nascent peptidoglycan biosynthesis in the presence or absence of pknAMtb-overexpression. Early-log phase cells of M. smegmatis (KMS2) containing pCK314 were divided into two flasks, and pknAMtb was expressed in one of the flasks for 2 hr by adding 0.1% of acetamide. Subsequently, both cultures were incubated with tetracycline (20 ng ml-1) for 2 hr to express gfp-wag31Mtb and examined by a florescent microscope. For vancomycin staining, Van-Alexa568 (5 mg/ml) was added to each culture at the end of tetracycline induction and further incubated for 20 min before examining by a fluorescence microscope. Average GFP and Van-Alex568 intensity from cells with pknAMtb-overexpression relative to that of cells without pknAMtb-overexpression are shown at the bottom of each panel. (p-value for the difference in GFP signals = 1.00 X 10-6, and for Van-Alexa568 signals = 0.004). Bar, 5 μm.
