# Supplementary material for: Regulation of Polar Peptidoglycan Biosynthesis by Wag31 Phosphorylation in Mycobacteria
Source: BMC Microbiol. 2010 Dec 29;10:327. doi: 10.1186/1471-2180-10-327 (PMC3019181; doi:10.1186/1471-2180-10-327)
Supplement: Additional file 4 — Table A2: Primers used in this study. List of primers used to make plasmid constructs for this study. [file 1471-2180-10-327-S4.DOCX]

**Additional file 2 (Table A2).** Primers used in this study.

| **Primer Name** | **Sequence** |
| --- | --- |
| Ngfp-wag-1 | 5’-TGGCTAGCAAAGGAGAAGAAC-3’ |
| Ngfp-wag-2 | 5’-GCAGGTGTAAGCGGCATATCCATGCCATGTGTAATCC-3’ |
| Ngfp-TBwag-3 | 5’-ATGCCGCTTACACCTGCCGA-3’ |
| Ngfp-TBwag-4 | 5’-CTAGTCTAGACTAGTTTTTGCCCCGGTTGAA-3′ |
| WagYTHF | 5’-CACTGAATTCATGCCGCTTACACCTGCC-3’ |
| WagYTHR | 5’-GTCAGGATCCCTAGTTTTTGCCCCGGTTG-3’ |
| GFPWag-Bam-5 | 5’-C ATGGCTAGCAAAGGAGAAGAA-3’ |
| GFPWag-Cla-Spe-3 | 5’-TATATCGATACTAGTCTAGTTTTTGCCCCGGTTGA-3’ |
